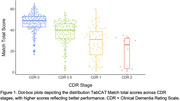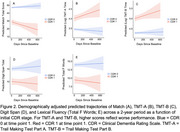# A Good Match for the Uniform Dataset? Comparison of the Digital TabCAT Match Test with Traditional Tests of Attention and Executive Function for Monitoring Disease Severity

**DOI:** 10.1002/alz70857_104684

**Published:** 2025-12-25

**Authors:** Alexandra J. Weigand, Elena Tsoy, Kelly J. Atkins, Sabrina Jarrott, Claudio Reck‐Rivera, Tiffany R. Brailow, Katherine L. Possin

**Affiliations:** ^1^ Memory and Aging Center, University of California San Francisco, San Francisco, CA, USA

## Abstract

**Background:**

Efficient digital cognitive assessments may offer an alternative to traditional paper‐and‐pencil tests. We evaluated the concurrent and predictive validity of the TabCAT Match test compared to Uniform Dataset (UDS) attention and executive function tests.

**Method:**

TabCAT Match is a 2‐minute measure of executive function and speed that requires participants to rapidly touch the simple picture corresponding to a number from 1 to 7 on an iPad. TabCAT Match and UDS Trail Making Test (TMT‐A and ‐B; total time, log transformed), Digit Span Total, and Fluency (total ‘F’ words) from 441 older adults evaluated at the UCSF Alzheimer's Disease Research Center were analyzed, 100 with 2‐year follow‐up data. Receiver operating characteristic area under the curve (AUC) bootstrapped comparisons and ANCOVAs adjusting for age, sex/gender, educational attainment, race, and testing language were used to compare Match v. the UDS tests on classification of Clinical Dementia Rating Scale (CDR) stages and subjective cognitive decline (SCD). Additionally, longitudinal trajectories were compared using linear mixed effects models adjusting for the same demographics.

**Result:**

Match scores differed across all CDR stages assessed (CDR 0–2; Figure 1), whereas only some UDS tasks differed between CDR 0 and 0.5 (TMT‐A & ‐B, Fluency), CDR 0.5 and 1 (TMT‐A and ‐B), and CDR 1 and 2 (none). Match differed between cognitively unimpaired (CU) and SCD in unadjusted models, but no tasks differed between these groups in demographically adjusted models. AUCs were numerically highest for Match in differentiating CDR 0 CU vs. CDR 0 SCD, all CDR 0 (CU + SCD) vs. 0.5, CDR 0.5 vs. 1, and CDR 1 vs. 2, although AUCs were only statistically different from TMT‐A, Digit Span, and Fluency for CDR 0 vs. 0.5. Participants with mild dementia (CDR 1) had a significant decline on Match and TMT‐A over a 2‐year period relative to CDR 0 participants, with no differences observed for TMT‐B, Digit Span, or Fluency (Figure 2).

**Conclusion:**

The TabCAT Match test appears to perform equally well or superior to traditional paper‐and‐pencil UDS measures for differentiating and monitoring disease severity, with the added advantage that it is self‐administered and takes only 2 minutes to complete.